# Role of Sphingosylphosphorylcholine in Tumor and Tumor Microenvironment

**DOI:** 10.3390/cancers11111696

**Published:** 2019-10-31

**Authors:** Mi Kyung Park, Chang Hoon Lee

**Affiliations:** College of Pharmacy, Dongguk University, Seoul 04620, Korea; cutejoyer@hanmail.net

**Keywords:** inflammatory, lysosphingolipids, neurological, sphingosylphosphorylcholine, tumor microenvironment

## Abstract

Sphingosylphosphorylcholine (SPC) is a unique type of lysosphingolipid found in some diseases, and has been studied in cardiovascular, neurological, and inflammatory phenomena. In particular, SPC’s studies on cancer have been conducted mainly in terms of effects on cancer cells, and relatively little consideration has been given to aspects of tumor microenvironment. This review summarizes the effects of SPC on cancer and tumor microenvironment, and presents the results and prospects of modulators that regulate the various actions of SPC.

## 1. Introduction

Sphingosylphosphorylcholine (SPC) is a unique lysosphingolipid produced from sphingomyelin. Although SPC is found in the blood and is reported to be released from platelets [1], increased SPC levels are observed in various disease states, such as ascites of patients with ovarian cancer, atopic dermatitis, Niemann Pick disease (NPD), and metabolic syndrome [1,2,3,4,5]. Accordingly, SPC affects the pathophysiology of these and various other diseases. SPC also has a variety of effects on the cardiovascular system, central nervous system, immune system and skin, as well described in the literature [6,7]. 

Recent research highlights the involvement of SPC in cancer progression [6,8,9,10,11,12]. While the tumor microenvironment is emerging as a key contributor to malignant progression and metastasis, research on the role of SPC in cancer has been mainly focused on the characteristics originated from cancer cells, such as proliferation, apoptosis, and cancer cell migration.

However, even though SPC affects blood vessels, nerves, inflammation, and immunity, few reviews about the influences of SPC on the tumor microenvironment have yet been published. In addition, only a few reports cover the substances that mimic or block the various actions of SPC.

Therefore, in this report, we review the effects of SPC on cancer and the tumor microenvironment, and related mechanisms, to clarify the future research directions, including trends in the development of modulators that regulate SPC action.

## 2. Occurrence and Mechanism of Action of SPC 

**Figure 1:**
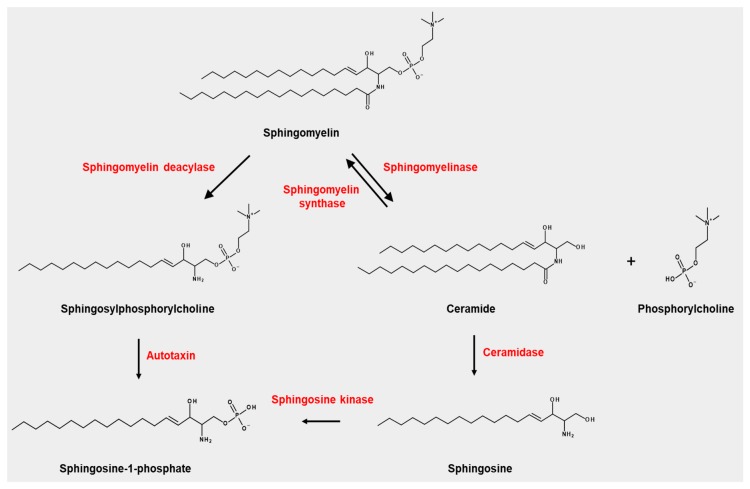
Occurrence of sphingosylphosphorylcholine (SPC) and its related metabolisms.

SPC is a lysosphingolipid produced by the sphingomyelin deacylase from sphingomyelin, a route that is not yet fully elucidated, and another unidentified pathway [13,14] (Figure 1). Certain diseases, for example, NPD type A, which exhibit pathologic SPC accumulation in different organs, are characterised by a lack of acid sphingomyelinase activity [3].

SPC can be degraded by autotaxin, producing sphingosine-1-phosphate (S1P), which is a well-studied sphingolipid that displays potent biological activity via S1P receptors (S1P1–S1P5). However, the production of S1P from SPC by autotaxin is not a major route for S1P.

This section briefly summarizes the excellent literature reviews about the occurrence and mechanism of action of SPC, and mainly adds new findings that have been discovered since [6,7].

Sphingosylphosphorylcholine (SPC) is produced from sphingomyelin by sphingomyelin deacylase. Autotaxin can degrade SPC into sphingosine-1-phosphate, which is also produced from sphingosine by sphingosine kinase. Sphingomyelin is also degraded by sphingomyelinase into phosphorylcholine and ceramide, which is converted into sphingosine by ceramidase. Ceramide is converted into sphingomyelin by sphingomyelin synthase.

### 2.1. SPC Occurrence and Metabolism

The levels of SPC in cells, tissue or plasma, are generally low under normal conditions [3,15]. SPC does occur naturally in plasma [15]. The concentration of SPC in plasma of an average person was determined as 50 nM and increased in serum (estimated to 130 nM) [15]. This suggests that SPC may be produced from activated platelets albeit at a low level. Dramatically higher levels of SPC are found: in lesions in patients with atopic dermatitis; in the brain in patients with NPD type A; malignant ascites in ovarian cancer patients; and in cerebrospinal fluid (CSF) from patients with subarachnoid hemorrhage (SH) [2,13,16]. As such, SPCs from affected lesions in atopic dermatitis patients cause a scratching response in murine models [17]. 

Strong positive correlation occurred between SPC and CD163 in plasma from metabolic syndrome patients [5]. NPD is an inherited metabolic disease in which sphingomyelin accumulates in lysosomes of cells [3]. SPC accumulated remarkably in the brain of two patients with NPD type A (830 and 430 pmol/mg protein in 27-month-old and 16-month-old children with severe and milder neurological status, respectively) [3]. In contrast, no significant increase in SPC occurred in brain tissue from a patient with a 3.5-years history of NPD type B [3]. However, SPC is elevated in dried blood spots of NPD type B patients [18].

Median plasma SPC level was significantly elevated in NPD type C by 2.8-fold. For miglustat-naïve NPD type C patients, aged 2–50 years, the area under the receiver operating characteristics curve was 0.999 for SPC [19]. In particular, SPC, along with other lipids such as lysosphingomyelin-509 and urinary bile acid metabolite 3β-sulfooxy-7β-N-acetylglucosaminyl-5-cholen-24-oic acid, can be an excellent marker for the diagnosis of NPC type C [20]. 

To overcome the difficulty of SPC detection, which requires the use of expensive instruments such as liquid chromatography-tandem mass spectroscopy, aptamer for SPC detection was screened and applied to the development of a highly sensitive enzyme-coupled aptamer assay system for SPC detection [21]. This was the first example using aptamers in the detection of SPC and helped SPC monitoring in practical situations.

Sphingomyelin deacylase is involved in the production of SPC. This enzyme expression is increased in lesions in patients with atopic dermatitis [13]. Sphingomyelin deacylase produces SPC from sphingomyelin [22], but its relative amounts in other cell types under normal conditions are not determined, and its activity in lymphocytes is much lower than in skin cells [22]. SPC production in cardiac myocytes was remarkably enhanced by endothelin-1 [23], which occurred via breakdown of sphingomyelin, suggesting the participation of sphingomyelin deacylase. Until now, the expression of sphingomyelin deacylase has not been characterized in the heart. 

While some knowledge about production of SPC has been reported, very little is known about the subsequent metabolism of SPC. However, SPC (10 μM) injected into blood in the guinea-pig heart was undetectable after a single pass though the coronary circulation, suggesting rapid metabolism [15]. Choline-specific glycerophosphodiester phosphodiesterase hydrolyzed SPC efficiently [24]. Human neutral sphingomyelinases such as nSMase1 and nSMase2, can hydrolyze SPC efficiently under detergent-free conditions [25]. Another candidate enzyme for SPC metabolism is autotaxin, which has been involved in control of tumor cell migration [26]. Autotaxin exist in human plasma [27] and can hydrolyze SPC to sphingosine-1-phosphate [28].

### 2.2. Similarities and Differences between SPC and Lysophosphatidylcholine 

SPC belongs to lysophospholipid, along with lysophosphatidylcholine (LPC) which is found in humans with the highest amount of hundreds μM [29]. Both are recognized as substrates of autotaxin, which hydrolyses SPC and LPC to produce S1P and LPA, respectively [28]. SPC acts as a ligand for S1P receptors (S1P1–S1P3) and LPA receptors (LPA1–LPA3), where S1P and LPA act as ligands for S1P receptors and LPA receptors, respectively [30]. LPC and SPC can reduce organ injury and dysfunction [31], act as antigens against NK cells [31] and activate ecto-5′-nucleotidase in human umbilical vein endothelial cells [32].

Unlike LPC, SPC inhibits the expression of BACE1 [33]. In addition, SPC specifically binds to calmodulin as a calmodulin inhibitor, whereas LPC does not [34]. SPC reduces the rate at which RyR channels open, but LPC reduces the rate at which these channels close [35]. LPC increased ROS generation in Jurkat T cells [36], induced chemotaxis of CD16 + NK cells [37], and showed a therapeutic action in an experimental sepsis animal model [38]. In contrast, SPC had none of these effects, but it induced differentiation of mesenchymal stem cells into SMC-like cells but LPC did not [39], and the release of Ca^2+^ via GPR12 [40], whereas LPC did not. LPC activates Ca^2+^ release from intracellular stores by binding to GPR55 [41]. It is not yet known how SPC affects GPR55. For more details on the physiological and pathophysiology of LPC, please see recent reviews [42,43].

### 2.3. Mechanism of SPC Action

In this part of the paper, we introduce targets and signaling pathways to mediate the effects of SPC. First, we mention the direct target of SPC and then molecules affected by SPC.

#### 2.3.1. SPC and G Protein-Coupled Receptors (GPCRs)

GPCRs may be involved in SPC action, and this suggestion has been evaluated in several reports using PTX.

##### G Protein-Coupled Receptor 3 (GPR3) and G Protein-Coupled Receptor 12 (GPR12)

GPR3 and GPR12 are well described in reviews by Laun et al. and Kostenis [44,45]. GPR3 and GPR12 have recently been recognized as novel targets of cannabidiol [44]. We will only cover GPR3 and GPR12 related to SPC. 

GPR3 and GPR2 constitutively activate Gαs (without its ligand), increasing the basal level of intracellular cAMP [44,46,47]. However, SPC and S1P can act as ligands to activate GPR3 and GPR12 [40,48].

GPR3 is highly expressed in various neurons and is also found in other organs, such as lung, kidney, liver, ovary and testes, but its expression is also elevated in the brain of patients with dementia [44,49]. GPR3 is needed to maintain meiotic arrest in porcine oocytes and rodent oocytes through pathways implicated in the regulation of cAMP and cGMP [50,51]. SPC, a ligand of GPR3, inhibits oocyte maturation.

GPR12 was found in the neurons of the amygdala, frontal cortex, hippocampus, hypothalamus, olfactory bulb, piriform cortex and thalamus regions of the brain, and in mRNAs of testes and oocytes [44]. GPR12, which can be activated by SPC, regulates cellular activities, such as survival, proliferation, neurite extension, cell clustering, keratin reorganization, and maintenance of meiotic arrest [52,53,54,55]. GPR3 and GPR12 are also engaged in neurite cAMP signaling and outgrowth in rat cerebellar granule neurons [51].

##### G Protein-Coupled Receptor 4 (GPR4) and Ovarian Cancer G Protein-Coupled Receptor 1 (OGR1)

GPR4 is a GPCR that emerged as a receptor of SPC and then turned out to be a proton-sensing GPCR. However, GPR4 might mediate the effects of SPC on endothelial cells [56]. GPR4 can block ERK activation, independent of a ligand [57]. Recently, encouraging reports outlined the importance of the GPR4 gene itself in diseases, independent of SPC. GPR4 is also involved in the progression of head and neck cancer, epithelial ovarian cancer (EOC) and colorectal cancer [58,59]. A positive correlation between GPR4 expression and a higher microvascular density in EOC was observed, but this was not evident in benign ovarian tumor tissues [60]. GPR4 expression and microvascular density in EOC are strongly associated with lymph node metastasis and clinical stage. Moreover, GPR4 is reported to block the spread of B16F10 melanoma cells [61]. GPR4 also plays a significant role in the regulating of intestinal inflammation, as was identified in inflammatory bowel disease models using mice lacking GPR4 [62].

OGR1, like GPR4, was also initially reported to be a ligand for SPC but turned out to be a proton- sensing GPCR. Interestingly, SPC antagonized proton-sensing OGR1, resulting in reduced accumulation of inositol phosphate and cAMP [63]. 

##### S1P Receptors

SPC acts as a ligand of the S1P receptor at a higher concentration than S1P [64,65]. EC50 values for S1P2 and S1P3 receptors are known to be 100 nM, which is normally present in serum [15]. These S1P receptors can couple with Gi, Gq, G12, among others, to regulate adenylate cyclase, PLC and Rho, for example, located in downstream signalling pathways [66].

Details of the signalling process through the S1P receptor by SPC are described in Nixon et al. [7]. SPC’s actions through the S1P2 receptor include YAP dephosphorylation, phosphorylation of serine 71 in vimentin and α-smooth muscle actin expression [67,68,69].

Actions via the S1P3 receptor include the protection of ischemic and post-ischemic myocardial tissue, and MCP-1 production [70,71].

#### 2.3.2. Calcium Related Molecules

##### Calmodulin/Ryanodine receptors (RyR)/ Sphingolipid Ca^2+^ Release-Mediating Protein of the Endoplasmic Reticulum (SCAMPER)

Ca^2+^ is released from the endoplasmic reticulum, mitochondria and lysosome. SPC activated Ca^2+^ release from the endoplasmic reticulum, leading to acute elevations in cytosolic-free Ca^2+^ [72,73]. Sphingosine triggered the release of Ca^2+^ from two-pore channel 1 located on the surface of lysosomes [74]. High concentrations of SPC (10–20 μM) and sphingosine (30–50 μM) promoted the release of Ca^2+^ from the sarcoplasmic reticulum, and low concentrations of sphingosine inhibited Ca^2+^ release [75].

Calmodulin (CaM) is an intracellular receptor for SPC, which raises the possibility of novel, endogenous regulation of CaM [34]. CaM modulates hepatic membrane polarity by PKC-sensitive steps in the basolateral endocytic pathway [76]. SPC displaces CaM from its targets on cerebral microsomes, leading to CaM inhibition [77]. SPC has been reported to regulate ryanodine receptors (RyR) through interaction with CaM [77].

SPC can bind directly to the cytoplasmic side of RyR [78]. SPC derived from plasma membrane activates cardiac RyR channels [35]. SPC also releases Ca^2+^ from cardiomyocyte by acting as a ligand for SCAMER [79].

#### 2.3.3. Keratin 8 (K8) Phosphorylation, Reorganization and Epithelial Mesenchymal Transition (EMT) 

##### Transglutaminase-2 (TGase-2)

Transglutaminase-2 (TGase-2) is a Ca^2+^-dependent enzyme that forms an isopeptide bond by cross-linking the lysine ε-amino group between γ-carboxamides of glutamine residues [80]. SPC activates transglutaminase in human keratinocytes, but it also increased TGase-2 expression in a pancreatic cancer cell line, PANC-1 [81,82]. This SPC-induced TGase-2 enhanced the phosphorylation of serine residue 431 in keratin reorganization by JNK [82].

##### Epithelial Membrane Protein 2 (EMP2)

Epithelial membrane protein 2 (EMP2) belongs to the tetraspan protein superfamily and is involved in cell adhesion through integrin binding [9]. SPC reduces the expression of EMP2 in A549 cells, which caused SPC-induced K8 phosphorylation and reorganization [9]. K8 phosphorylation can also be achieved with JNK and ERK. During this process, the interaction of alpha4 protein and caveolin-1 appears to decrease the expression of protein phosphatase 2A [9].

##### Rheb Like-1 (RhebL1) Protein and YdjC Chitooligosaccharide Deacetylase Homologue (YDJC)

*Rheb like-1 (RhebL1)* is a member of the Ras superfamily of G proteins, and mammals harbor two Rheb genes: *Rheb1* and *RhebL1 (Rheb2)* [11,83]. SPC increases RhebL1 and YDJC to participate in the keratin reorganization process [11]. In particular, K8 phosphorylation and reorganization by SPC-induced RhebL1, a small G protein, was achieved through Akt1 activation due to binding of Akt1 to RhebL1 [11].

YdjC chitooligosaccharide deacetylase homologue (YDJC) is a member of the YDJC family, and this enzyme can deacetylate acetylated carbohydrates in the degradation of oligosaccharides [84].

SPC can induce expression of YDJC in A549 lung cancer cells [8]. SPC-induced YDJC also induces phosphorylation and reorganization of K8, leading to enhanced migration and invasion [8]. YDJC can induce EMT through ubiquitination of PP2A, especially through binding to cell division cycle protein 16 (CDC16) [85].

##### ERK2/Thrombospondin-1 (TSP-1)

Thrombospondin-1 (TSP-1) belongs to a family of secreted glycoproteins, and TSP-1 binding to latent TGF-β1 complex can convert latent TGF-β1 to its biological active form in fibrotic renal disease and experimental diabetic nephropathy [86,87]. SPC seems to be involved in EMT of mammary MCF10A cells. In particular, promoting secretion of TSP-1 appears to be an explained mechanism of EMT induction [88]. ERK2 seems to be involved in the increased secretion of TSP-1 by SPC.

#### 2.3.4. Signaling Molecules and Other Unclassified Molecules

##### Hippo Signaling

The Hippo-signaling pathway regulates organ size through proliferation and apoptosis [89]. SPC can regulate the Hippo-signaling pathway, causing an activation of yes-associated protein (YAP) followed by inhibition [67]. However, even the strong SPC-induced effects seen in large tumor suppressor kinase 2 and YAP did not mediate the antiproliferative SPC response [67]. 

##### Fyn and Focal Adhesion Kinase (FAK) in Stress Fiber Formation

Fyn belongs to Src-family of kinases, the first proto-oncogenes to be identified [90]. The effect of SPC on Fyn has not been studied in cancer cells, but mainly in fibroblasts. For example, Fyn acts downstream of SPC to stimulate stress fiber formation via ROCK in fibroblasts [91]. SPC enhances stress fiber formation through activation of Fyn/RhoA/ROCK signaling [92]. SPC also induces FAK activation in intact Swiss 3T3 cells [93]. 

## 3. Effects of SPC on Cancer Hallmarks from Tumor Itself

Cancer has about ten common features we call hallmarks of cancer [94], which can be divided into those originating from the cancer itself and those derived from the microenvironment around the cancer. If we find specific targets that regulate these hallmarks, this provides essential logic for the development of anticancer agents.

**Figure 2:**
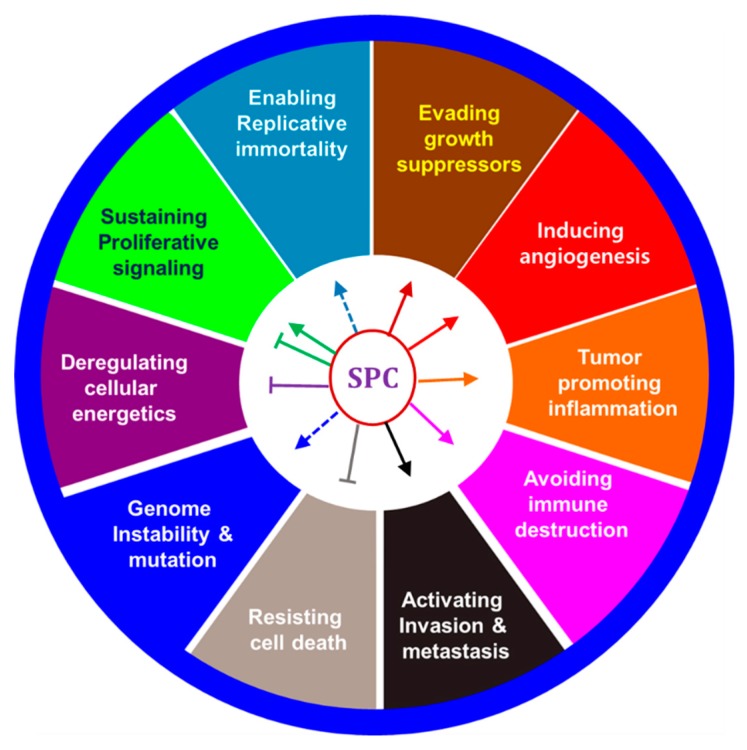
Effects of SPC on hallmarks of cancer.

SPC has been studied in several cancers and appears to inhibit or promote proliferation depending on various concentrations. Also, SPC can act as an inflammatory or anti-inflammatory factor, and it contributes to angiogenesis because of its vascular effects. Therefore, SPC effects were described in terms of ten cancer hallmarks (Figure 2), and the effects of SPC on neuronal elements in cancer progression, which are now being actively studied, are also discussed.

### 3.1. Effects of SPC on Proliferation and Apoptosis 

SPC inhibits proliferation in many cancer cells. For example, SPC (1–10 μM) induced apoptosis of MDA-MB-231 breast cancer cells via autophagy/Akt/p38 and the JNK pathway [10]. SPC increased intracellular Ca^2+^ in DU 145 and PC3 hormone-refractory prostate cancer cell lines, causing apoptosis [95]. SPC suppressed three out of four species of pancreatic cancer cells [96] but induced Swiss 3T3 fibroblasts regarding both DNA synthesis and cell proliferation [97]. SPC promoted cell-cycle progress at the G1/S phase in Swiss 3T3 fibroblasts but inhibited it in PANC-1 cells in reduced fetal bovine serum (FBS) concentrations [96]. In thyroid FRO cancer cells, SPC showed antiproliferative effects through G2/M phase arrest [98].

Treating neuroblastoma (neuro2a) cells with SPC resulted in activation of PKC-delta for SPC-induced apoptosis. A rapid translocation from cytosol to membrane and PKC-delta cleavage produced a fragment involved in apoptosis [99]. 

SPC (more than 10 μM) -evoked cell death was reported in non-cancer cells. For example, SPC-induced cell death of human adipose tissue-derived mesenchymal stem cells occurred via cytochrome c-dependent and caspase-3-dependent apoptosis pathways [100]. 

### 3.2. Effects of SPC on Invasion and Metastasis

Recently, metastasis and invasion of cancer cells has been actively studied with SPC, which regulates keratin reorganization, leading to enhanced migration of epithelial tumor cells [101,102,103]. MEK/ERK and JNK signaling controls SPC-induced phosphorylation and reorganization of K8 in human pancreatic and gastric cancer cells. Ser431 in K8 is the crucial residue whose phosphorylation is sufficient to induce keratin reorganization, leading to enhanced migration of human epithelial tumor cells. 

SPC induces invasion of MDA-MB-231 breast cancer cells via secretion of matrix metalloproteinase-3 and perinuclear reorganization of K8 filaments [11,12]. SPC evokes EMT and secretion of TSP-1 in MCF10A immortalized breast cells via ERK2 [88].

SPC-induced RhebL1 binds to Akt1 and activates Akt1, which is involved in keratin phosphorylation and reorganization induced by SPC, promoting the migration and infiltration of lung cancer cell lines [11]. Enhanced YDJC expression and loss of EMP2 expression are also associated with increased cell migration and invasion by SPC [9,103]. In particular, YDJC is involved in keratin phosphorylation and reorganization and EMT involving ubiquitination of PP2A by interaction with CDC16 [8,85]. The decreased expression of PP2A by SPC appears to be an upstream regulatory step that can explain the activation of ERK and JNK by SPC.

SPC induced phosphorylation of S71 in vimentin, leading to a reorganization of vimentin filaments of MDA-MB-435S human breast cancer cells [68].

### 3.3. Effects of SPC on Growth Suppressor 

There is not much research on the effect of SPC on p53, a growth suppressor in cancer cells. SPC evoked necrosis and autophagy but suppressed apoptosis in A549 and H157 cells via downregulation of Akt and mTOR complex 1 (mTORC1) [104]. Furthermore, SPC could not promote autophagy in p53-deficient cells. Thus SPC-induced autophagy in these cells was through downregulation of Akt/mTORC1 and induction of the p53 signal pathway. Additionally, there are results that show SPC regulates expression of p53 in human umbilical vein endothelial cells (HUVEC). Thus, the level of p53 in HUVECs deprived of FBS and fibroblast growth factor-2 was significantly enhanced, but SPC significantly inhibited the enhanced p53 level at 6 h [105].

### 3.4. Effects of SPC on Genome Instability and Mutation, Replicative Immortality

ROS can kill cancer and increase the heterogeneity of cancer cells by inducing genome instability. [106]. SPC reduced both the mitochondria membrane potential and ROS levels in A549 cells [104]. There have been many reports that SPC induces ROS in tissues and non-cancer cells (keratinocytes, MS1 pancreatic islet endothelial cells, intrapulmonary arteries) [107,108,109,110]. Therefore, further studies are expected on the effects of genome instability, mutations, and related mechanisms for SPC-induced ROS in cancer cells. It has been reported in HeLa cells that genomic instability is increased by overexpression of NOX1 involved in ROS increases [111]. NOX5 NAD(P)H oxidase also enhances growth of DU 145 prostate cancer cells [112].

Replicative immortality is a vital characteristic of cancer cells, and there are no studies on the effect of SPC on telomere or telomerase. However, the important molecule for controlling expression of telomerase is MYC transcription factor [113]. Interestingly, SPC can enhance the DNA binding of upstream stimulating factor, one of the necessary basic helix-loop-helix-zipper protein, by which c-MYC mRNA was rapidly increased, reaching maximum levels at 0.5–1 h, and additionally increased after 12 h [114]. These results suggest that SPC might regulate the expression of telomerase through the expression of c-MYC.

### 3.5. Effects of SPC on Cancer Metabolism

The importance of cancer metabolism has been newly highlighted, opening a new chapter in cancer treatment [115,116,117]. Limited research has been conducted on the effects of SPC on cancer metabolism. SPC evoked a prolonged increase in basal pH, and SPC-evoked alkalinization was abolished in rat pituitary CHC cells treated with 2-deoxy-D-glucose, which depletes cellular ATP stores [118]. SPC may activate Na^+^-H^+^ exchange, mediated via an amiloride-insensitive exchange mechanism. However, it is unknown whether these SPC effects can inhibit or promote the development of cancer. GPR4 and OGR1 (related to SPC), which sense the acidic tumor microenvironment that results from cancer metabolism, regulate cancer cell proliferation and metastasis, inflammation including immune cell function, and angiogenesis [119]. Thus, although compelling, the answer to the question of how SPC affects cancer metabolism is still unknown.

## 4. Effects of SPC on Tumor Microenvironments

Regulation of the tumor microenvironment is emerging as an essential strategy in overcoming cancer heterogeneity since the tumor microenvironment itself contributes to cancer heterogeneity [120]. Noncancerous cells constituting tumor microenvironments include macrophages, dermal cells, vascular endothelial cells, and neutrophils, which promote or inhibit cancer [121]. It is, therefore, necessary to reeducate the microenvironment that promotes cancer to a microenvironment that inhibits cancer [122,123]. Recently, immune checkpoint inhibitors, which have been under the spotlight, were designed to attack cancer cells by reeducating immune cells in the tumor microenvironment [124]. Among the hallmarks of cancer, ‘inducing angiogenesis’, ‘promoting inflammation’, and ‘avoiding immune destruction’ are related to the tumor microenvironment. Additionally, the neural contribution to cancer progression was recently included in the tumor microenvironment.

### 4.1. Effects of SPC on Angiogenesis

Tumor growth and metastasis is dependent on angiogenesis and lymphangiogenesis, evoked by signals released from tumor cells [125,126]. As mentioned earlier, SPC has various effects on constituent cells of the cardiovascular system, such as VSMC and endothelial cells. SPC induces wound healing, and angiogenesis is a necessary step for proper wound healing. For example, SPC induces chemotactic migration of human and bovine endothelial cells equal to the response that was exerted by vascular endothelial cell growth factor (VEGF) [127]. SPC also induces mRNA expression associated with angiogenesis in cryopreserved transplanted human fat tissues [128]. Moreover, SPC has been associated with rapid downregulation of Edg1, a S1P-specific receptor involved in endothelial cell chemotaxis [127]. 

SPC can induce a Gi-dependent transactivation-mediated phosphorylation of VEGF receptor 2 [56], which is needed for SPC-induced Akt activation. ERK is also activated by SPC via a GPR4-independent pathway. Therefore, both ERK and Akt are implicated in the angiogenic effects of SPC on endothelial cells. 

uPA expression is increased at transcriptional and translational levels in the SPC-induced angiogenesis of HUVEC cells. SPC increased the activity of cell-surface-associated PA, too [129]. Moreover, SPC dose-dependently increased the activity of ecto-5’-nucleotidase, the primary enzyme regulating extracellular adenosine production in HUVECs [32].

Above a specific concentration, SPC may have an opposite effect. For example, at a concentration of ≥10 μM, SPC promotes apoptosis of vascular endothelial cells. Thus, it can induce apoptosis through ROS-mediated activation of ERK at concentrations ≥10 μM through a caspase-3 dependent pathway [109].

### 4.2. Effects of SPC on Inflammation

Inflammatory cells and mediators such as cytokines, chemokines and prostaglandins in the tumor microenvironment regulate many proinflammatory processes, which occur in an autocrine or paracrine manner [130,131]. SPC can stimulate intercellular adhesion molecule-1 (ICAM-1) in keratinocytes [132], IL-6 production in fibroblasts [133], and activation of dendritic cells to produce IL-12 [134]. SPC-induced IL-6 production is achieved by PKC and p42/44 ERK in fibroblasts [135].

In studies of diseases unrelated to cancer, many reports suggest that SPC may be associated with inflammation. Thus, it has been reported that SPC found in high concentrations in atopic dermatitis causes scratching and histamine, and Rho/ROCK signaling are implicated in SPC-induced scratching [17]. SPC also induced degranulation and plasma exudation of skin mast cells in mice [136].

SPC evokes itch-related responses through the production of leukotriene B4 (LTB4) from keratinocytes [137]. LTB4 is a leukotriene produced from arachidonic acid by the lipoxygenase pathway and is involved in inflammation [138]. These results suggest that an increase in SPC induces LTB4 -mediated itching in chronic dermatitis [139].

SPC can stimulate HUVECs, resulting in Jak/STAT3-, NF-κB-, and activator protein-1-mediated C-C motif chemokine ligand 2 production and expression of the ICAM-1 in HUVECs [140]. These molecules are deeply involved in the establishment of inflammatory events. SPC can increase the secretion of the monocyte chemoattractant protein-1 (MCP-1) from rat VSMC, and the increase of MCP-1 expression was confirmed in cerebral arteries [1]. These results suggested that SPC is a proinflammatory mediator in cerebral arteries.

SPC dose- and time-dependently upregulated IL-8 in ovarian cancer cells (HEY, OCC1, and SKOV3), indicating a potential role of SPC in tumor associated inflammation [141]. 

SPC also acts as an anti-inflammatory substance: it reduced inflammation of endotoxemia in rats [142]; and reduced IL-1β-induced prostaglandin E2 production in renal mesangial cells [143]. SPC, combined with HDL, shows anti-inflammatory action [144]. Several anti-inflammatory effects can be attributed to the presence of SPC in HDL [145].

### 4.3. Effects of SPC on Immune Evasion

Immunity may promote cancer or inhibit cancer growth. It is called immune evasion when cancer cells evade the immune function that kills cancer [94]. Some tumor cells evade immune surveillance by reducing the expression of antigen-presenting proteins at plasma membrane, rendering them to escape from cytotoxic T lymphocytes [146]. But more often, tumors secrete cytokines that prohibit effector T cell responses and stimulate suppressive regulatory T cells [147].

There is no direct study of the effect of SPC on immune evasion in cancer. However, results of studies on the effects of SPC on various immune cells may become a basis for study of the effects of SPC on immune evasion.

SPC promoted the proliferation of resting spleen cells [148], stimulated human monocyte-derived dendritic cell chemotaxis [149], increased expression of human leukocyte antigen-DR, CD86 and CD83, and enhanced the T-cell priming ability of dendritic cells. SPC can stimulate the production of IL-12 and IL-18 by dendritic cells and act as a potent chemoattractant for natural killer cells activated by IL-2-, IL-12- and IL-15- but not for interferon-α [37,134].

An acidic pH in the tumor microenvironment suppresses T cell responses. The proton-sensing GPCRs mentioned above, GPR4, OGR1 and G2A, are present in immune cells and may be involved in T cell responses in acidic tumor microenvironments. There is a possibility that SPC participates in cancerous immune modification as a regulator of such GPCRs.

### 4.4. Effects of SPC on the Neuronal Contribution to Tumor Growth

The importance of neuronal effects on cancer progression and the tumor microenvironment has been emphasized recently. For example, activation of sympathetic nerves due to stress promotes tumor, and the reduced density of tumor innervation resulted in higher recurrence-free survival [150,151]. The increased chemotherapeutic response to β-blockers is mediated via anticancer and anti-angiogenic activities [152]. 

Melatonin (N-acetyl-5-methoxy-tryptamine), known as a biological clock regulator, is implicated in the induction of apoptosis, cell cycle arrest, proliferation inhibition, and immune regulation [153]. Defects in the biological clock and activation of the sympathetic nerve system are induced by various interactions between neurological factors and tumor microenvironment resulting in cancer progression [154,155].

Studies investigating the regulation of tumor microenvironment have not considered the effect of nervous system on cancer cells. Therefore, it is vital to determine the role of neuronal interactions in biological clocks and tumor microenvironment, and reeducate the tumor microenvironment to facilitate antitumor activity. Signaling substances released from neurons mediate cancer malignancy via various pathways that increase neovascularization, metabolic activity, immunosuppression, cancer cell proliferation and metastasis.

SPC promotes the release of Ca^2+^. However, no reports exist about the high amount of SPC, or increased Ca^2+^ released in neurons, in the tumor microenvironment. Nevertheless, the action of SPC in brain and neuronal tissues and cells is well known. That is, as mentioned above, SPC induces Ca^2+^ release in various neurological tissues and cells, such as rat brain preparations and pituitary cells [118,156,157].

The effects of SPC on astrocytes contribute to the neurodegeneration of NPD. Incubation with SPC causes an increased intracellular Ca^2+^ in cultured astrocytes to release glutamate, producing a secondary intracellular Ca^2+^ increase in co-cultured neurons [158]. Repeated stimulation of this process might be a cause of cytotoxicity and neurodegeneration. Repeated exposure of astrocytes by SPC (mimicking the in vivo state) resulted in proliferation of astrocytes to release the inflammatory cytokine, TNF-α [158]. This cytokine could exert a neurodegenerative effect. More research is required to elucidate the role of SPC-induced effects on astrocytes in the pathogenesis of type ‘‘A’’ NPD. SPC alone induced the release of precursor and mature IL-1β (mIL-1β) from LPS-primed MG6 cells, possibly due to lytic functions [159]. However, SPC prohibited ATP-induced caspase-1 activation, followed by the release of mIL-1β [159]. 

## 5. Therapeutic Effects of SPC and SPC Antagonists

**Figure 3:**
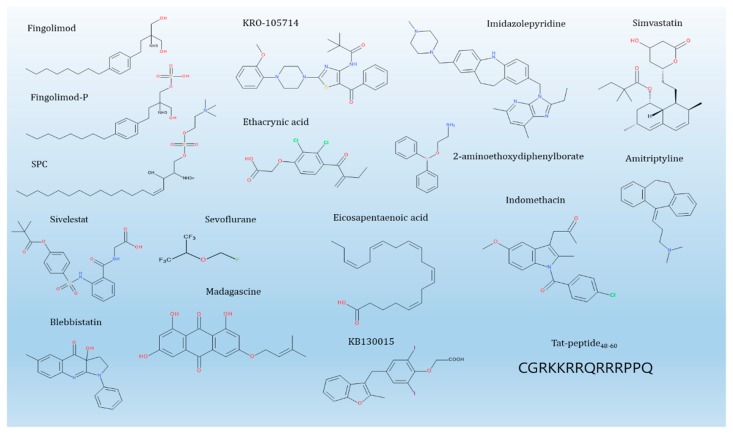
SPC and its blockers.

“5.1 Therapeutic Effects of SPC” summarizes the results of in vivo animal-level results that show direct therapeutic effects by SPC. In addition, “5.2 Therapeutic Effects of SPC blocker” introduced the effects of potential direct blocker of SPC, followed compounds that suppress SPC signaling or action (Figure 3).

### 5.1. Therapeutic Effects of SPC

SPC 2 μM intraperitoneally, daily for 14 days, attenuated hepatic damage in bile duct ligated rats with extrahepatic cholestasis by preventing oxidative stress and inflammatory processes [160]. SPC (10 mg/kg) alleviates inflammation and organ injury/dysfunction evoked by LPS in the rat [142]. Intravenous SPC (0.625–2.5 μg/g) protected ischemic and post-ischemic myocardial tissue in a murine model of myocardial ischemia/reperfusion injury. SPC also suppressed leukocyte adhesion to TNFα-activated endothelial cells and saved rat neonatal cardiomyocytes from apoptosis [70].

SPC 2 and 10 μM attenuated renal damage in rats with contrast nephropathy by blocking oxidative stress and apoptosis [161]. Prophylactic infusion of SPC (2, 10 μM) powerfully attenuated quisqualate-induced behaviors in rats and prevented neuronal degeneration. Moreover, SPC may be of clinical meaning in alleviating progressive group 1 mGluR-induced hippocampal cognitive and motor disorders, such as Alzheimer’s disease, brain seizure, and stroke [162].

### 5.2. Therapeutic Effects of SPC Blockers

Limited data, particularly in vivo data, are available about compounds that inhibit cancer by direct competition with SPC action. This might be because the direct target of SPC is not clear, and it may be unclear whether an SPC agonist or antagonist is useful for diseases.

However, there is a possibility that compounds directly suppress SPC. For example, fingolimod is a sphingosine-backbone molecule with immunological activity. It is marketed for the oral administration of patients with relapsing multiple sclerosis [163]. Sphingosine kinase 2-mediated fingolimod-phosphate functions as a functional antagonist for S1P receptors and SPC blockers (Figure 3) [55,164,165]. As such, fingolimod and fingolimod-phosphate, which have structural similarities with SPC, inhibited the SPC-induced phosphorylation and perinuclear reorganization of K8 [55]. GPR12 is involved in this process and appears to be accompanied by restoring expression of PP2A.

KRO-105714 [N-(5-benzoyl-2-(4-(2-methoxyphenyl)piperazin-1-yl)thiazol-4-yl)pivalamide] inhibits SPC-induced cell proliferation. It was discovered via high-throughput screening and had anti-inflammatory activity in atopic dermatitis [166]. 

Ethacrynic acid can inhibit SPC-induced phosphorylation and reorganization of K8, which inhibits TPCase-2 induced by SPC, preventing JNK from phosphorylating serine 431 [167].

There was also an approach to search soil microbes for substances that inhibit the migration of PANC-1 cells induced by SPC [168]. Although subsequent studies have not been reported, this suggests that increased migration by SPC could be used as a screening method to identify inhibitors of SPC action.

Furthermore, the acidic conditions of the tumor microenvironment are regarded as notable factors for the growth of cancer cells. Regulation of pH-sensing GPCRs, regarding related SPC activity, suggests the possibility of controlling these acidic conditions.

Imidazolepyridine, which is found by GPR4-mediated serum response element reporter assay, is a negative allosteric modulator of proton-sensing GPR4 prohibiting the GPR4/Gs protein-mediated but not the TDAG8/Gs protein-mediated cAMP response to acidic pH [169]. GPR4 modulator could not affect SPC-induced Ca^2+^ mobilization, a typical Gq/11 protein-mediated process. Hence, some anticancer agents may control the acidic tumor microenvironment created as a result of cancer metabolism.

Reports on substances that block SPC-induced phenomena in diseases other than cancer are relatively more frequent than in cancer. For example, 2-aminoethoxydiphenylborate inhibited SPC (1 μM)-induced Ca^2+^ sensitization in smooth muscle tissue obtained from urinary bladders [170]. However, this compound did not act as an SPC blocker but did act on a downstream pathway such as Rho kinase.

A similar effect, involving inhibition of Rho kinase, is inhibition of SPC by simvastatin. Indeed, simvastatin inhibited SPC-induced differentiation of HMSC into SMC by allaying RhoA/Rho-kinase-dependent activation of the autocrine TGF-β1/Smad2 signaling [171]. 

Amitriptyline inhibited the contractile responses of rat tracheal ring to SPC (10 μM): the concentration of amitriptyline (mean ± standard deviation) required to exert 50% inhibition (IC_50_) was 98.2 ± 21.8 μM, respectively [172].

Sivelestat induced concentration-dependent (1 ~ 300 μM) vasorelaxation in SPC (30 μM)-induced contraction of human gastric artery, but it could not induce vasorelaxation in conditions of high K^+^ (40 mM) depolarization. Sivelestat prohibited SPC-induced VSM contraction, but it did not affect Ca^2+^-induced contraction [173].

Blebbistatin (IC_50_ = 26.1 ± 0.2 and 27.5 ± 0.5 μM for GbaSM-4 and A7r5 cells, respectively) blocked the chemotaxis of vascular smooth muscle cells toward SPC (1 μM) [174]. Blebbistatin (IC_50_ = 22.8 ± 1.26 μM) also inhibited SPC-induced contraction of collagen-gel fiber populated by GbaSM-4 VSMC from the basilar artery of guinea pigs [175].

Sevoflurane (1.7%, 3.4% solution), but not propofol, inhibits Rho kinase-dependent contraction evoked by SPC (10 μM) in the porcine coronary artery [176].

Madagascine can be synthesized or isolated from several Rhamnus species, and has more potent biological activities than the parent compound emodin. Madagascine (10 μM) significantly relaxed the abnormal constriction in porcine VSM by SPC (30 μM) and the effect was blocked by compound C (20 μM), a cAMP-activated protein kinase inhibitor [177].

Eicosapentaenoic acid (EPA) inhibits SPC-induced Rho-kinase activation in vitro and in vivo SH models [178]. The inhibition of cerebral vasospasm induced by SPC or after SH by EPA suggests beneficial roles of EPA in the treatment of cerebral vasospasm [178,179]. In clinical trials based on these findings, EPA inhibited the cerebral vasospasm (CIV) after the onset of subarachnoid haemorrhage in a prospective, nonrandomized study. The occurrences of CIV (7% vs. 21%; *p* < 0.012) were significantly lower in the EPA than control group [180]. Omega-3 and omega-6 docosapentaenoic acid (60 μM) also inhibited SPC (30 μM)-induced Ca^2+^-sensitization of VSM contraction via suppressing Rho-kinase activation and translocation [181].

Tat-peptide inhibits AGC-family kinases (protein kinase B; serine/thereonine-protein kinase 1; ribosomal S6 protein kinase 1; mitogen- and stress-activated protein kinase 1), Ca^2+^/calmodulin-dependent protein kinase (CAMK)-family kinases (CAMK1, and maternal embryonic leucine zipper kinase), and an STE family kinase (mitogen-activated protein kinase, kinase 1) [182]. In HeLa cells, Tat-peptide prohibited phorbol ester-evoked ERK1/2 phosphorylation, suggesting that Tat also suppressed PKCs. In thyroid cells, Tat-peptide alleviated SPC-evoked Ca^2+^-fluxes depending on PKC [182].

SPC (100 nM) induced the current of G protein-coupled inwardly-rectifying potassium channel in atrial myocytes. KB130015, a new drug structurally related to amiodarone, added during superperfusion with SPC, inhibited the induced current with the same potency as for the current induced by acetylcholine [183].

Indomethacin markedly inhibited the diuretic and natriuretic effect of SPC (30 μg/kg/min) [184].

Substances that directly block the action of SPCs in other cells, but not cancer cells, should not be neglected for study as anticancer drugs. In particular, such substances can provide useful information for considering which steps to suppress when SPC cannot be directly inhibited. Such substances can also expand their uses as therapeutic agents for cancers related to SPC, through drug repositioning based on blocking the action of SPC.

## 6. Perspectives

We have summarized the effects of SPC on cancer characteristics and, in doing so, sought directions for studying the role of SPC in cancer progression. There are many studies about the effects of SPC on specific cancer hallmarks; however, some hallmarks have not yet been studied. In particular, the role of SPC in angiogenesis, inflammation, and nerves were studied in non-cancer cells, and are now the basis for future research into the tumor microenvironment when assessing the effects of SPC on cancer development. Specifically, assessment of the effect of SPC on acidic-sensing GPCR and the variation and action of GPCR in the cancer microenvironment could be fascinating and influential.

Additionally, there are cases where SPC characteristics show contradictory results, depending on dosage, and it is necessary to distinguish whether the reason is due to SPC properties per se or SPC binding with lipoprotein. Therefore, careful research designs are required, considering physiological and pathological conditions in the presence or absence of FBS, serum deprivation, and the SPC concentrations needed for specific experiments. There is also a need to conduct more extensive research to determine the amount of SPC in various in vivo pathological models or human tissues. If fluctuations in SPC amounts are not well defined, attention should also focus on differences in the degree of expression of the various genes considered to be targets for SPC and to the possibility of mutation.

SPC agonists, including SPC itself, and SPC blockers have revealed potential slowly. Nonetheless, the use of SPC blockers in cerebral haemodynamic disorders and SPC-related cancers seems to be a possibility. The development of SPC-associated proton-sensing GPCR modulators will also provide new opportunities for regulating the acidic microenvironment, and results of SPC agonists and blockers will provide valuable information about the regulation of these GPCRs. In addition, results of SPC agonists and blockers will offer crucial information about the regulation of these GPCRs.

In conclusion, research involving SPC in cancer is of immense importance and could soon provide new opportunities if breakthroughs are made.
